# Pathogen genomics in healthcare: overcoming barriers to proactive surveillance

**DOI:** 10.1128/aac.01479-24

**Published:** 2024-12-05

**Authors:** Alexander J. Sundermann, Rossana Rosa, Patrick N. A. Harris, Evan Snitkin, Waleed Javaid, Nicholas M. Moore, Mary K. Hayden, Krisandra Allen, Kyle Rodino, Sharon J. Peacock, Lilian M. Abbo, Lee H. Harrison

**Affiliations:** 1 Microbial Genomic Epidemiology Laboratory, Center for Genomic Epidemiology, University of Pittsburgh 6614 , Pittsburgh, Pennsylvania, USA; 2 Department of Epidemiology, School of Public Health, University of Pittsburgh 171669 , Pittsburgh, Pennsylvania, USA; 3 Infection Prevention and Control Program, Jackson Health System 23214 , Miami, Florida, USA; 4 Centre for Clinical Research, Faculty of Medicine, The University of Queensland 303224 , Herston, Queensland, Australia; 5 Herston Infectious Disease Institute, Metro North, QLD Health 157827 , Herston, Queensland, Australia; 6 Central Microbiology, Pathology Queensland, Royal Brisbane and Women's Hospital 3883 , Herston, Queensland, Australia; 7 Department of Microbiology and Immunology, University of Michigan Medical School 12266 , Ann Arbor, Michigan, USA; 8 Division of Infectious Diseases, Department of Medicine, University of Michigan Medical School 12266 , Ann Arbor, Michigan, USA; 9 Division of Infectious Diseases, Department of Medicine, Icahn School of Medicine at Mount Sinai 377569 , New York, New York, USA; 10 Departments of Internal Medicine and Pathology, Rush University Medical Center 2468 , Chicago, Illinois, USA; 11 Digital Epidemiology Services Inc, San Francisco, California, USA; 12 Department of Pathology and Laboratory Medicine, Perelman School of Medicine, University of Pennsylvania 192716 , Philadelphia, Pennsylvania, USA; 13 Department of Medicine, Addenbrooke’s Hospital, University of Cambridge 151904 , Cambridge, England, United Kingdom; 14 Division of Infectious Diseases and Miami Transplant Institute, University of Miami Miller School of Medicine 698249 , Miami, Florida, USA; 15 Division of Infectious Diseases, University of Pittsburgh School of Medicine 271847 , Pittsburgh, Pennsylvania, USA; Johns Hopkins University School of Medicine, Baltimore, Maryland, USA

**Keywords:** genomic epidemiology, surveillance, outbreaks, infection prevention

## Abstract

Pathogen genomic surveillance in healthcare has the potential to enhance patient safety by detecting outbreaks earlier, thereby reducing morbidity and mortality. Despite benefits, there are barriers to adoption, including cost, expertise, and lack of standardized methodologies and incentives. This commentary advocates for 1) investment from healthcare payors, public health, and regulatory bodies and 2) additional research on genomic surveillance for improving patient outcomes and reducing infections. Effective implementation will require strategic investment and cross-sector collaboration.

## COMMENTARY

## LANDSCAPE OF WHOLE GENOME SEQUENCING FOR INFECTION PREVENTION AND CONTROL

In the ever-evolving domain of healthcare, the introduction of whole genome sequencing (WGS) has emerged as a transformative tool. Its invaluable applications in medical research are well recognized, but its role in infection prevention and control (IP&C) strategies has become increasingly crucial in enhancing patient safety (1). The ability of WGS to identify genetically related patient pathogens—indicative of potential transmission or a common source—provides IP&C departments in healthcare institutions with precise information necessary for timely interventions to halt outbreaks. Traditionally, due to high costs and infrastructure requirements, WGS is currently mainly utilized for reactive sequencing to confirm suspected outbreaks (2). This approach misses many outbreaks. Moreover, outbreak definitions and detection methods are non-standardized (3).

**Fig 1:**
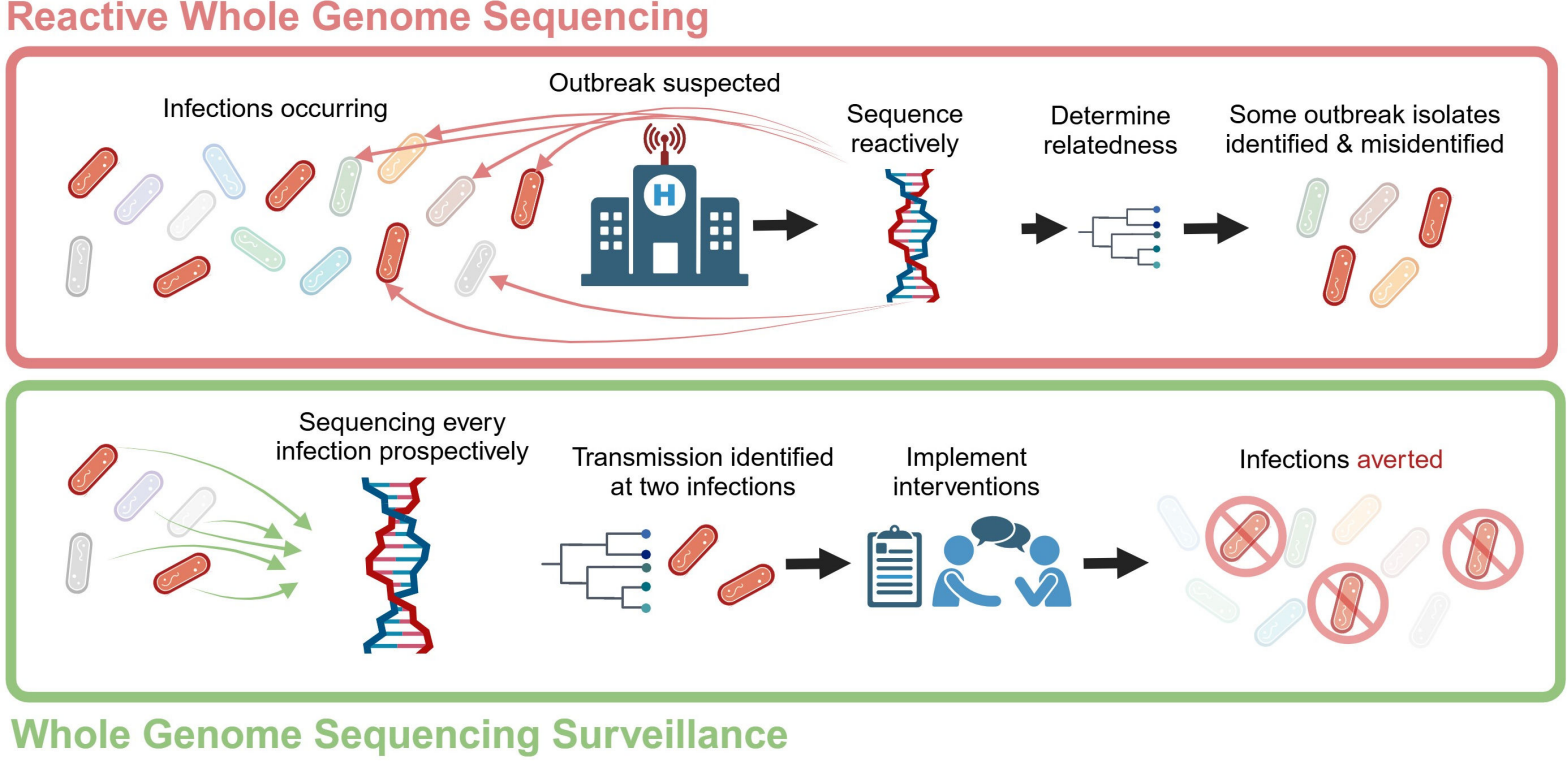
Difference in approach for reactive versus surveillance whole genome sequencing. Figure made with BioRender.

The concept of WGS surveillance, sequencing pathogens irrespective of the presence of a suspected outbreak, is not new (4). In recent years, there has been growing evidence supporting the use of WGS surveillance over reactive methods (Fig. 1). This approach has the potential to revolutionize our perspective on patient safety in IP&C, especially considering its capability to detect early and identify otherwise undetected outbreaks.

Despite its clear advantages in enabling early outbreak detection and preventing transmission, the shift from reactive WGS to the adoption of proactive pathogen WGS surveillance remains limited in healthcare. This commentary explores the barriers and solutions for making WGS surveillance a routine, proactive tool in clinical healthcare settings. A key issue is whether the current body of evidence is sufficient to engage and persuade healthcare leaders and payors to invest human and financial resources that will allow healthcare institutions and their patients to reap the benefits. Does the existing research adequately demonstrate the value of WGS surveillance, or is there a need for more comprehensive studies? These questions and many others are vital in understanding and overcoming the barriers to broader adoption in the healthcare sector (Table 1).

**TABLE 1:** Summary of barrier areas and potential steps forward

Area	Current status for genomic surveillance	Considerations	Future directions
Evidence	Multiple studies showing surveillance finds previously unrecognized outbreaks and with interventions can reduce infections and deaths	Causal evidence in reducing HAIs Differences in fungal, viral, or bacterial approaches Cost-effectiveness	Additional, independent analysis showing benefits and outcomes Detailed modeling analysis for single centers and broader communities
Financial	Studies showing support for net cost-savings by averted infections	Different healthcare institution types In-house versus outsourced capacity Consistent funding opportunities Reliable reimbursement model	Analysis on impact by institution type Listing of resources available to institutions
Infrastructure	Variability in access and knowledge for genomics	Necessary equipment and laboratory space Dedicated staff Competencies in analysis Trained infection prevention departments capable of making interventions	Analysis on optimal practices Programs to train individuals on analysis and interventions
Methods	No consensus on optimal methodology for genomics	Consistent methods across multiple centers Bioinformatic pipeline methods Identifying genetically related pathogens	Professional society guidance on optimal analysis methods
Legal & Ethical	No guidance or professional society documents on legal, ethical, or regulatory guidelines	Data security, privacy, and storage Considerations in patient disclosure equitable access	Discussions and analysis on best practices
Regulatory		Laboratory certification Development of billing or reimbursement models	Discussions with regulatory bodies on potential future pathways

## ASSESSING CURRENT EVIDENCE

In the field of infection prevention, interventions are selected based on evidence that they enhance patient safety. For WGS surveillance to become a standard practice, it must be similarly shown to reduce infections. The body of prospective studies examining the efficacy of this approach remains limited (5). Presently, the evidence suggests that WGS surveillance can provide earlier detection and identify outbreaks that remain undetected by conventional infection prevention methods, many of which are mischaracterized by traditional approaches (5). However, the critical question remains: Does this methodology actually lead to a reduction in infections following intervention? Establishing the effectiveness of interventions is challenging and necessitates the use of a counterfactual scenario to estimate the impact of intervention in preventing additional infections (Fig. 1). Retrospective evidence robustly supports the notion that many outbreaks remain undetected without genomic surveillance and progressed without intervention.

When considering WGS surveillance across various pathogen types—fungal, viral, and bacterial—the current evidence predominantly supports the utility of bacterial WGS. There have been a few studies to date that have examined the impact of WGS surveillance (Table 2). A study conducted at UPMC Presbyterian Hospital in the US has demonstrated the capability of WGS surveillance to detect multiple bacterial outbreaks of significant impact that were otherwise missed. Prior analyses at this center were retrospective, focusing on WGS surveillance without subsequent interventions (6). In a shift to a real-time, prospective approach, their findings indicated that interventions halted outbreaks in 95% of instances on the targeted route and prevented 62 infections over a 2-year period (7). This center conducted WGS across a variety of pathogens over several years, tracking transmission to different patients. The investigators found that even with the cost of WGS surveillance, their hospital had net savings of $695,706, a three-fold return on investment, in the prevention of infections during the same time period.

**TABLE 2:** Notable bacterial WGS surveillance studies

Author	Year	Pathogen	Duration	Outbreaks or notable findings	Hidden reservoirs uncovered	Interventions used
Forde et al.	2023	Multiple MDR bacteria	4 years	76 clusters	Multiple outbreaks, one focused on contaminated burn baths	Isolating affected patients, enhanced cleaning of high-risk areas, and changes in hospital policies regarding patient movement
Lee et al.	2023	Carbapenemase-producing *Enterobacterales*	8 years	Nine outbreaks linked to the environment	Contaminated medical equipment and outpatient facilities	Enhanced cleaning, isolation of affected patients, and process audits
Ludden et al.	2021	*Escherichia coli*	6 months	10 clusters, 20 patients; both carriage and clinical infection transmission	Patient mixed strain carriage	None used in real time, but promoted use to reduce bacteremia
Miles-Jay et al.	2023	*Clostridioides difficile*	9 months	Low levels of transmission, indicating successful IP&C practice	None as focused on asymptomatic carriers of *C. difficile* as potential sources of transmission	Routine screening for *C. difficile* carriage on admission and during hospitalization
Sherry et al.	2022	Multiple MDR bacteria	15 months	31% acquired MDRO in hospital, directed IP&C interventions	Uncovered unexpected transmission links between patients	Enhanced cleaning, isolation of affected patients, and patient screening
Stribling et al.	2023	*Pseudomonas aeruginosa*	10 years	Protracted outbreak	Persistent reservoirs identified in the hospital plumbing system, with pathogen persistence traced back to the 1990s	Enhanced cleaning of environmental surfaces, particularly the plumbing system
Sundermann et al.	2022	Multiple bacteria	2 years	99 outbreaks, 297 patients	Outbreaks from unsterile contrast preparation that followed manufacturer’s guidelines and an outbreak from contaminated gastroscopy, among others	Study was performed retrospectively, although interventions were made on one outbreak, including notifying the manufacturer of the medical equipment
Sundermann et al.	2024	Multiple bacteria	2 years	172 outbreaks, 476 patients	Outbreaks had been intervened upon with smaller endoscope outbreaks; found outbreaks originating at outside facilities	Enhanced cleaning, staff education, patient screening, and environmental sampling

Similarly, investigators in Brisbane, Australia initiated a real-time, prospective WGS surveillance project across three healthcare centers, focusing on multi-drug resistant organisms (8). Their findings revealed that WGS surveillance identified several ongoing transmission events, enabling a more precise allocation of intervention resources. Moreover, some institutions, such as the Walter Reed Army Institute of Research, have focused on specific pathogens like *Pseudomonas aeruginosa*, conducting retrospective and prospective WGS surveillance over multiple years (9). This targeted approach proved effective in slowing transmission by uncovering hidden reservoirs that traditional methods would likely overlook. Another recent study from Cambridge University Hospitals National Health Services Foundation Trust in England observed *Escherichia coli* transmission among colonized and infected patients using WGS surveillance (10). This approach uncovered multiple nosocomial outbreaks of *E. coli* where patients became colonized and subsequently infected with genetically related strains.

A recent analysis from investigators in the Royal Prince Alfred Hospital in Sydney, Australia investigated the real-time, prospective use of WGS surveillance on carbapenemase-producing *Enterobacterales* (CPE) over 8 years (11). The hospital identified multiple outbreaks linked to environmental isolates, including plasmid transfer, over the study period. The investigators stated that the identification of the source of some of these CPE outbreaks or detection of the outbreak entirely would not have been possible without WGS surveillance. The authors describe how they were able to use these findings to approach hospital administration for internal support to sustain the program. Another study performed in Melbourne, Australia examined the impact of prospective WGS surveillance within eight hospitals over 15 months (12). They sequenced 2,275 isolates and found that 31% of pathogens were transmitted. In this context, WGS uncovered numerous previously unrecognized transmissions, which led to targeted interventions. Their study highlighted the potential of WGS to significantly enhance infection prevention efforts by identifying transmission events that would have otherwise gone undetected.

WGS surveillance has also been helpful in determining the lack of isolate relatedness and arguing against nosocomial transmission, thus avoiding false alarms and resource misallocation. In a study from Rush University Medical Center, investigators performed WGS surveillance on *Clostridioides difficile* in an intensive care unit for over 9 months (13). The authors found very low transmission rates, giving support that current infection prevention practices are effective at preventing transmission, and efforts would be better focused on antibiotic and diagnostic stewardship. Moreover, only one out of 67 patients colonized on admission was linked as the source of a nosocomial case.

Given the prolonged duration of many bacterial outbreaks, which can extend over several months or even years, there is a window for reactive sequencing and interventions to halt the spread. Conversely, viral outbreaks characterized by shorter incubation and transmissibility periods necessitate rapid response times. Even a turnaround time of 14 days would be a delay that may possibly compromise the effectiveness of interventions for viral outbreaks, where the index patient’s transmissibility may have already passed, and any interventions would be ineffective. Nevertheless, the coronavirus disease (COVID-19) pandemic has given significant attention to the genomic surveillance of severe acute respiratory syndrome coronavirus 2 (SARS-CoV-2) (14). Additionally, the genomic surveillance of fungal pathogens poses unique challenges due to their eukaryotic genomes, resulting in limited studies focused on their role in hospital outbreak detection. However, WGS has proven useful when studying the introduction and spread of emerging pathogens, such as *Candida auris* (15). Adoption of these pathogen types in WGS surveillance programs will require additional evidence showing that it is both feasible and impactful.

## FINANCIAL CONSIDERATIONS

In evaluating the role of WGS surveillance in reducing hospital-acquired infections (HAIs), it is crucial to assess cost-effectiveness. Literature reviews and other analyses have suggested that WGS surveillance, especially for bacterial pathogens, may result in cost savings for both individual healthcare facilities and the healthcare system at large (16–18). Yet, these conclusions are primarily drawn from modeling studies that estimate the effectiveness of interventions or predict the number of outbreaks and transmissions averted within healthcare settings. The variability in sequencing costs influenced by each center’s access to resources also complicates the generalizability of these evaluations.

Determining the most accurate model for calculating 'cost savings' from preventing infections during an outbreak presents several challenges. First, the cost of sequencing per isolate may be variable depending on available resources (19). Second, should the calculation include the costs avoided in treating the infection, the reduced length of hospital stays, or other factors? How should the incentives for reducing reportable HAIs, as offered by the Centers for Medicare & Medicaid Services (CMS), factor into these calculations? Finally, the impact of these interventions on individual patient costs must be considered. Comparing these financial implications across various types of healthcare facilities—from tertiary care and academic centers to rural hospitals and long-term care facilities—further adds to the complexity. A potential solution could be the implementation of a multi-center cluster randomized trial across diverse healthcare settings. This approach would allow for the measurement and control of key variables, such as infrastructure, infection control practices, and genomic sequencing capabilities, thereby providing a more comprehensive understanding of the factors influencing the success of proactive surveillance.

The decision to conduct sequencing in-house or outsource it—to public health labs or industry partners, for instance—also impacts financial considerations. Despite the potential for higher costs using commercial laboratories, an increasing number of companies are entering the WGS field, suggesting a need for a detailed analysis to identify the most efficient approach.

At the crux of offering a sustainable WGS surveillance program is reliable financial support. Some academic healthcare institutions that are developing WGS surveillance methodologies are supported by research grants, but this approach is not sustainable. While public health programs received WGS funding for SARS-CoV-2, the funding frequently undergoes a ‘boom and bust’ cycle (20). In addition, many hospitals are financially stressed, particularly in the wake of the COVID-19 pandemic, and are, therefore, unable or unwilling to support the costs needed for a WGS surveillance program. Healthcare facilities must rigorously evaluate the available evidence and consider the potential missed opportunities for identifying and intervening in multiple outbreaks. Incentives from payors and/or regulatory agencies are required to make WGS surveillance more widespread.

When evaluating strategies to encourage the adoption of WGS surveillance from a financial standpoint, it is essential to identify who stands to gain the most from its implementation. A healthcare facility operates within a fixed budget and may not immediately recognize the direct cost savings associated with WGS surveillance in outbreak prevention, as these savings are primarily realized through reduced treatment expenses. However, the private or public payors responsible for covering treatment costs are likely to see significant financial benefits due to the decreased need for extensive interventions. Therefore, a dual approach that targets both healthcare facilities and their payors could be the most effective strategy. By highlighting the long-term financial advantages for payors and the potential for improved patient outcomes, this approach could foster greater collaboration and investment in WGS surveillance across the healthcare system.

## INFRASTRUCTURE REQUIREMENTS

Healthcare facilities considering the deployment of an effective WGS surveillance program must navigate multiple infrastructure requirements. A primary decision involves choosing between conducting sequencing in-house or outsourcing to a third-party commercial laboratory or public health service. For facilities opting for in-house sequencing, adequate laboratory capacity and space are essential to accommodate the equipment necessary for sequencing. Furthermore, if a facility decides to perform sequencing internally, it is imperative to have staff who are thoroughly trained in the sequencing process, from sample preparation to the resulting bioinformatics analysis. Determining the optimal number of employees required for the volume of sequencing undertaken is another critical aspect of infrastructure planning and can vary greatly depending on the level of automation included in the laboratory workflow. This calculation must account for the technical demands of sequencing operations and the analytical expertise needed to interpret the data generated, considering that, at this point, this is not a “billable” operation generating hospital revenues. Automated solutions could help overcome many of these barriers by offering services that streamline sequencing processes and minimize the need for extensive expertise in the preparation, implementation, and analysis of WGS. These solutions could be developed and implemented through a variety of approaches, ensuring flexibility and adaptability across different healthcare settings. Such services could be seen in examples, such as the use of matrix-assisted laser desorption ionization-time of flight (MALDI-TOF) for pathogen identification, in which commercial machines have automated the identification of various pathogens.

Beyond the technical execution of sequencing, it is crucial to have an IP&C department capable of understanding and acting on the WGS results. Infection preventionists must possess the skills and tools to investigate the causes of potential outbreaks effectively and orchestrate timely interventions. Without a thorough understanding to interpret results, IP&C department interventions may be misguided or perhaps may not intervene on actionable results. Ensuring these capabilities requires comprehensive training that covers all aspects of the sequencing process and its application in infection control.

The successful implementation of WGS surveillance extends beyond a single department or individual; it necessitates a multidisciplinary effort that brings together various experts to analyze data, generate insights, and execute interventions aimed at enhancing patient safety. Therefore, the infrastructure of a healthcare facility must support such collaboration, fostering an environment where seamless communication and coordinated action can occur. Establishing a streamlined pipeline for these multidisciplinary efforts is essential for the timely and effective use of WGS surveillance in improving patient outcomes.

## METHODOLOGICAL AND INTERPRETATIONAL CHALLENGES

Currently, the field lacks a consensus on a standardized approach for performing WGS, and given the diversity of sequencing technologies and bioinformatics pipelines, it is unlikely that a single method will be universally adopted. Instead, the focus should be on establishing standardized quality metrics and outcome assessments, allowing for comparison across different methods and platforms. This would enable a cross-institutional analysis without prescribing a specific methodology, similar to proficiency testing in clinical microbiology labs, where different tests can be used as long as the correct result is achieved. While many facilities depend on publicly available bioinformatics pipelines, others employ proprietary pipelines, which must undergo rigorous validation to ensure that they can accurately identify patient-related infections and transmission events, regardless of the specific pipeline used. Identifying the optimal criteria for selecting isolates for sequencing is another critical factor. Should a healthcare institution sequence isolates for every infection, or only those potentially associated with healthcare transmission? Should colonization isolates be sequenced? With resources often limited, prioritizing certain pathogens and types of infections, akin to practices in diagnostic stewardship, may be necessary, or such as selecting isolates from patients with hospital stays >2 days. Yet, if the costs associated with WGS decrease, broader sequencing may become feasible.

The debate over the effectiveness of long-read versus short-read sequencing for determining genetic relatedness further complicates the methodological choices in WGS. Both approaches offer advantages and disadvantages, but institutions must weigh these against their ability to accurately and confidently identify genuine outbreaks (21). Interpretational challenges also arise in defining what constitutes an outbreak. Single-nucleotide polymorphisms (SNPs) are commonly used to assess the genetic similarity between pathogens, yet bioinformaticians vary in the SNP thresholds they set to define transmission events (5). What one facility deems a transmission event might not be recognized as such by another, underscoring the need for professional guidelines to establish the criteria for identifying transmission or some level of certainty of transmission. Additionally, how should a healthcare facility determine plausible transmission between patients with genetically related isolates? There are vast differences in this approach, ranging from relying on same-time overlap on a healthcare unit to considering invasive procedures, healthcare workers, and the broader community (5). How should a healthcare facility respond to genetically related isolates with no common link? If no link is found, then simply nothing can be intervened upon. Therefore, facilities should thoroughly investigate all potential links (e.g., healthcare workers, procedures, contaminated equipment, and shared units) to identify these transmission events.

Moreover, the rapid evolution of bioinformatics and sequencing technology necessitates a continuous evaluation of new advancements and their implications for WGS. Facilities must consider how novel bioinformatics pipelines and sequencing technologies, such as metagenomics, affect the quality of DNA reads and overall results. This constant technological advancement requires the field to remain agile, ready to adapt to changes that can impact the accuracy and efficacy of WGS surveillance.

## LEGAL AND ETHICAL CONSIDERATIONS

Ensuring the security and privacy of data generated through WGS surveillance is paramount for both patients and healthcare facilities. For WGS surveillance to be an effective tool, it is essential for healthcare facilities and public health institutions to be able to share and analyze data across various entities while safeguarding protected health information. Currently, a universally optimal method for collaboration and data sharing across the pathogen WGS ecosystem does not exist (22).

**Fig 2:**
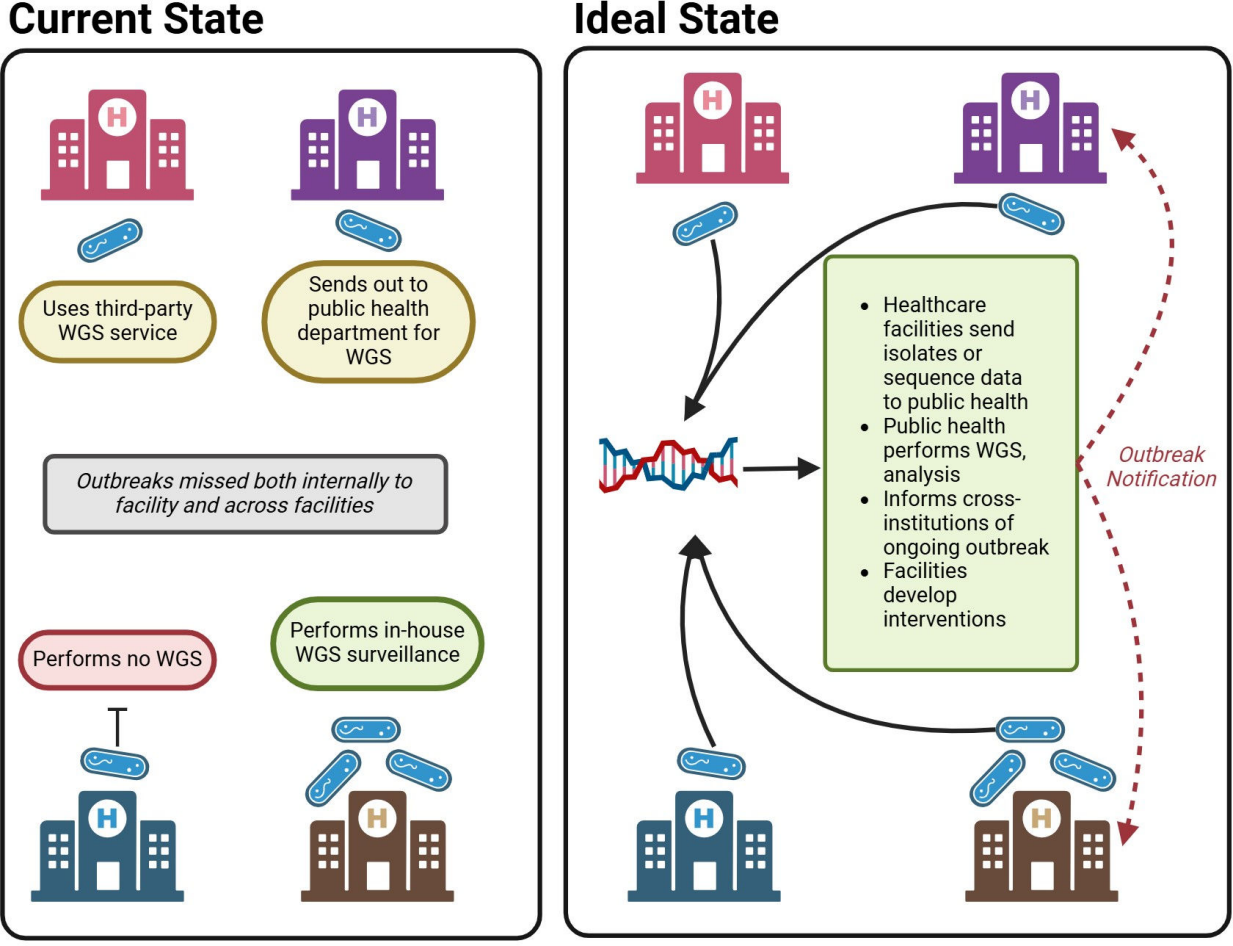
Current and possible scenario for the expanded whole genome sequencing surveillance system for healthcare facilities. Figure made with BioRender.

Questions arise regarding how one facility can compare genomic results with another and whether the evaluation of transmission across healthcare facilities should be the responsibility of public health departments. In its current state, even reactive WGS capacity is not common across healthcare facilities and health departments. Many facilities may have no access to WGS, while others may have resources from public health departments or even be mandated to send certain pathogens to public health departments for WGS (23). A fully developed and expanded healthcare WGS surveillance system may be similar to that of PulseNet. The success of PulseNet, where the CDC accesses genomic and patient-level data to manage foodborne outbreaks, offers a model for managing the interfacility transmission of HAIs, ensuring patient privacy through public health oversight. Facility-level WGS data could be securely shared with public health agencies for analysis and directed interventions (Fig. 2). Such an approach may be better apt to detect larger, cross-institutional outbreaks similar to the recent contaminated artificial tears outbreak, in which the CDC played a key role in monitoring sequence data, detecting the initial signals, and responding (24, 25). However, the variable funding and infrastructure of public health departments may limit their capacity to assume this role (26). For the successful detection of transmission, facilities must be committed to openly and promptly sharing their genomic data. While healthcare facilities might be reluctant to share data that could reveal outbreaks within their own walls, such transparency is crucial for preventing and controlling larger-scale outbreaks. This necessitates a shift toward recognizing data sharing as a standard practice, which carries privacy concerns. However, we can look back to PulseNet as a successful example in which large amounts of genomic data can be shared and still traced back to individual patients to provide follow-up and investigation.

Additionally, patient notification in the event of outbreak involvement warrants a careful consideration. The Council for Outbreak Response: Healthcare-associated Infections and Antimicrobial-resistant Pathogens (CORHA) offers guidelines on disclosing outbreak involvement to affected stakeholders, including healthcare staff and patients (27). These were developed when WGS was primarily reactive under the assumption that outbreaks were infrequent. However, as evidence suggests that outbreaks may be more common than previously understood, the strain on healthcare resources for patient notification increases. This raises questions about whether patients should be informed of their genetic linkage to infections in other patients in the absence of a known transmission route (because these may represent false alarms) and whether healthcare facilities are obligated to report ongoing outbreaks within their own institutions.

Lastly, equitable access to WGS surveillance is critical. Efforts must ensure that facilities in low-resource settings may have access to similar levels of resources for WGS surveillance as their counterparts in more resource-developed areas to prevent discrimination against the populations they serve. This may involve the establishment of funding mechanisms and training to support equitable access to WGS technologies (28).

## REGULATORY LANDSCAPE

The involvement of regulatory bodies is crucial in healthcare, particularly when implementing WGS surveillance. It is essential for laboratories to adhere to standards, ensuring they perform clinical and microbiological tests accurately and reliably. For all clinical microbiology labs, compliance with the Clinical Laboratory Improvement Amendments (CLIA) regulations is the bare minimum in the United States and establishes the requirements for laboratory testing, including tests incorporated into patient records that influence clinical care. The necessity for healthcare facilities conducting WGS for infection prevention purposes to obtain CLIA certification raises significant considerations given the extensive process involved in securing such certification. While CLIA regulates diagnostic tests for clinical care, questions arise regarding whether it is necessary for whole genome sequencing that is used for IP&C and may not be considered direct patient care. While not formally surveyed, some US-based health departments are requiring their own laboratories to have CLIA certification to perform WGS. During the initial stages of the COVID-19 pandemic, however, CMS provided guidance allowing non-CLIA-certified labs to perform WGS and share the data with public health departments for outbreak detection purposes, with the stipulation that results could not be used for patient diagnosis or treatment decisions (29). However, for laboratories functioning under a CLIA certification, the validation of sequencing approaches is a critical step to ensure accuracy, reliability, and regulatory compliance before these methods can be applied for epidemiologic purposes. This validation process ensures that the sequencing technologies meet the high standards required for clinical use and provides confidence in the results used for outbreak detection and infection control (30).

Furthermore, the roles of the Food and Drug Administration and CMS in the context of WGS must be evaluated. Laboratory tests develop and utilize current procedural terminology codes for the appropriate billing of patients and insurance providers. As WGS becomes more widely used, it might similarly require integration into this billing framework. One possible approach is to allow reimbursement for the use of whole genome sequencing for species identification for patient diagnosis/treatment and incidentally use the data for transmission analysis. Additionally, there is an established precedent for reporting HAIs to CMS. What initially began as an incentive program for facilities to report their HAIs has evolved into a penalty system for those with HAI rates surpassing a predetermined threshold. A comparable mechanism could be envisioned for WGS surveillance, encouraging facilities to report transmission events and achieve a specific sequencing coverage of their infections or colonizations, ensuring that transition to a more sensitive HAI detection method, and therefore increased reporting, is not misinterpreted as poor performance. A similar approach was seen with the development of antimicrobial use and resistance reporting to CMS (31). This proposal, however, introduces several complex considerations and intricacies not fully explored in this paper.

## FUTURE RESEARCH DIRECTIONS

Several promising research avenues could significantly deepen our understanding and refine the implementation of WGS surveillance. First, a critical area of future inquiry involves establishing more causal evidence that WGS surveillance effectively reduces HAIs following targeted interventions. This evidence base would solidify the role of WGS as a pivotal tool in infection prevention and control strategies. The analysis approach for this method is difficult, and institutions cannot simply examine reduction in the size of outbreaks or percent of isolates that are related. When an outbreak is detected, and an intervention is enacted, the aim is to stop the outbreak on the intervened route. However, an outbreak may still grow, for example, in other areas of the hospital where there had been no interventions to stop the spread of transmission. In a scenario of retrospective WGS surveillance with no intervention versus prospective WGS surveillance with interventions, an institution may see equal outbreak sizes, but effective interventions to stop the transmission. Given this, the most promising approach may be to evaluate the subsequent spread of an outbreak on the same transmission route that was intervened upon.

Building on this, there is a pressing need for detailed clinical and economic impact modeling to quantify the potential benefits of WGS surveillance on healthcare facilities and the broader healthcare system. Such studies provide valuable insights into the cost-effectiveness of WGS initiatives, guiding resource allocation and policy development.

As the fields of machine learning and artificial intelligence (AI) continue to advance, there is a vast potential for these technologies to enhance the utility of WGS data (32). Future research should investigate how machine learning and AI can improve the accuracy of outbreak detection, automate data analysis processes, and refine intervention strategies. These technological advancements could revolutionize the way we identify, track, and manage infectious disease outbreaks.

Additionally, the ethical and legal dimensions of WGS surveillance demand thorough exploration. Key issues include patient consent, data privacy, implications of WGS data on patient care decisions, and ensuring equitable access to WGS resources across different populations and healthcare settings. Patient consent is generally not required because there is no contact with patients, just the use of existing EHR data and isolates that are collected for routine clinical and/or infection prevention purposes. Regardless, addressing these concerns is crucial for fostering trust, ensuring compliance with regulatory standards, and promoting the fair use of WGS surveillance technologies.

To date, there has been no professional society or group to formally address the strategic use of WGS or WGS surveillance in healthcare settings. Societies, such as the Society for Healthcare Epidemiology of America (SHEA), the Association for Professionals in Infection Control and Epidemiology (APIC), the American Society for Microbiology (ASM), the Infectious Diseases Society of America, the European Society of Clinical Microbiology and Infectious Diseases, or others should have a role in the creation of such guidelines. Additionally, professional groups, such as CORHA or the Consortium for Clinical Metagenomics in Infectious Diseases, have multidisciplinary experts available to provide input and guidance on such measures. Collaborations among these institutions may greatly benefit the broader adoption of WGS surveillance.

## CONCLUSIONS

WGS surveillance offers the potential for substantial improvements in patient safety, contingent on strategic investment. Achieving this requires commitment from hospital leadership, public health agencies, and regulatory bodies funding public health efforts. The transformative impact of multidisciplinary genomic surveillance initiatives on detecting and managing outbreaks suggests a promising pathway for a similar paradigm shift in healthcare patient safety. WGS surveillance represents a largely untapped resource with the potential to markedly improve patient outcomes, decrease infection rates, and save lives.
